# Increased heart rate on first day in Intensive Care Unit is associated with increased mortality

**DOI:** 10.12669/pjms.326.11507

**Published:** 2016

**Authors:** Duygu Kara, Seda Banu Akinci, Gulcin Babaoglu, Ulku Aypar

**Affiliations:** 1 Dr. Duygu Kara, MD, Anesthesiology and Reanimation Unit, Hacettepe University Medical Faculty, Ankara, Turkey; 2 Prof. Dr. Seda Banu Akinci, MD, Anesthesiology and Reanimation Unit, Hacettepe University Medical Faculty, Ankara, Turkey; 3 Dr. Gulcin Babaoglu, MD, Anesthesiology and Reanimation Unit, Hacettepe University Medical Faculty, Ankara, Turkey; 4 Prof. Dr. Ulku Aypar, Anesthesiology and Reanimation Unit, Hacettepe University Medical Faculty, Ankara, Turkey

**Keywords:** Intensive care unit, Maximum heart rate, Mortality

## Abstract

**Objective:**

To investigate the association of maximum HR during the first day of intensive care unit (ICU) and mortality.

**Methods:**

Data of 850 patients over 45 years of age, who were hospitalized in ICU, was retrospectively analyzed. They were divided into two groups; Group-I, patients with maximum HR<100/min Group-II, patients with maximum HR≥100/min on first day. The groups were compared regarding age, sex, use of beta-blockers, use of inotropic and vasopressor drugs, hemodynamic parameters, anemia, mechanical ventilation, length of hospitalization (ICU and total), mortality (ICU and total), and CHARLSON & APACHE-II scores.

**Results:**

The mean age of patients was 63±12 years and 86% were after non-cardiac surgery. Maximum HR was 83±11 in Group-I and 115±14/min in Group-II (p=0.002). Group-II patients had more frequent vasopressor and inotropic drugs usage, (p<0.001), anemia, mechanical ventilation (p<0.005), higher CHARLSON & APACHE-II scores, stayed longer in ICU and hospital, and had higher ICU and hospital mortality compared to group-I (p<0.05). APACHE-II scores and maximum HR<100/min were independent variables predicting ICU mortality in multivariate logistic regression analysis whereas usage of beta-blockers was not.

**Conclusions:**

Our study showed that maximum HR less than100/minute during the first day of ICU is associated with decreased mortality in Intensive Care Unit.

## INTRODUCTION

Heart rate (HR) depends on the patient’s mood, physical activities, ventilation type, body temperature, myocardial contractility and myocardial capacity.^1^,^2^ During critical illnesses, patients have an increased metabolic rate in all types of organ metabolism. Maximum HR has been shown to indicate severity of disease and increased short-term mortality in critically ill patients.^3^ Cardiovascular diseases and other medical conditions have an increased risk of long-term mortality due to tachycardia, experienced among critically ill patients as a result of increased sympathetic activity, insufficient pain control or sedation, arrhythmias or pro-arrhythmogenic treatments.^4^-^6^

It is difficult to define a threshold value for HR because it must be individualized in the context of the patient’s overall hemodynamic status and any pre-existing comorbidities.^7^ Numerous large, epidemiological studies have shown that elevated HR is an independent risk factor for mortality and morbidity in even healthy individuals with or without hypertension and in patients with CAD, myocardial infarction (MI), or congestive heart failure (CHF).^8^ Several large placebo-controlled trials have demonstrated that drugs which reduce HR, including beta blockers, can reduce mortality and morbidity in patients with acute MI or CHF.^9^

It has been suggested that tight control of HR can improve outcomes in the ICU in different clinical settings, such as perioperatively during non-cardiac surgery and after myocardial infarction, sepsis, or perioperative stroke. Beattie et al. defined maximal HR as the upper range of HR and reported that in trials in which patients receiving beta blockers had a maximal HR of less than 100/minute there was a significant reduction in the incidence of postoperative MI, whereas in trials in which patients had maximal HR more than 100/ minute, there was no reduction in MI and the cardioprotective efficacy of perioperative beta blockers can be improved by achieving HR control.^10^ Ischemic events have been shown to occur when HRs increase to 100/min.^11^ In large cohorts of patients with acute MI, admission HR>90-100/min has been associated with approximately 3-fold higher mortality compared with an admission HR<60–70/min.^12^

As described above, previous studies in potentially overlapping clinical settings have used different HR targets. Therefore, although intensivists are becoming more HR conscious, the target HR limit in a mixed population of critically ill patients is unclear. In this study, we hypothesized that a maximum HR of <100/ minute within 24 hours of admission to a mixed ICU would be associated with decreased mortality.

## METHODS

Following ethical approval from the Hacettepe University Faculty of Medicine Interventional Non-Clinical Research Ethics Committee, files of all patients aged ≥ 45 years, since these were expected to have more comorbidities than younger patients, and who had been hospitalized in Anesthesiology and Reanimation ICU in the previous five years, were analyzed retrospectively. Patients were grouped according to diagnosis before and during ICU hospitalization, and surgical procedures. Patients’ systolic (SBP) and diastolic (DBP) blood pressures, mean arterial pressure (MAP) on admission, cardiac, vasopressor and inotropic drug use during hospitalization and HRs were recorded. All beta blockers used were recorded. Hypotension was defined as <90/60 mmHg, and if anemia was identified, the lowest hemoglobin value was recorded from the hospital laboratory database. The type of ventilation was also recorded if mechanical ventilation (MV) was needed in that period.

Patients were classified into two groups according to maximum (≥ 100/min, >5 min measurement) HR within 24 hours of ICU admission. This maximal HRs were without beta blockade. Group I was constituted from patients with maximum HR<100/min, while Group II was constituted from patients with maximum HR≥100/min. Admission diagnosis, medical history, concomitant relevant risk factors, mortality scores (Charlson and APACHE II), cardiac, inotropic and vasopressor drugs administered during the ICU stay were recorded for all patients.

All analyses were performed using SPSS (Statistical Package for Social Sciences, version 15.0). Normal distribution of the variables was assessed using the Kolmogorov-Smirnov test. One-way ANOVA or Student’s t test were used to compare groups in terms of normally distributed quantitative variables. The Kruskal-Wallis and Mann-Whitney U tests were used to compare the groups in terms of nonparametric data. The paired t-test or Wilcoxon tests were used to compare repeated measurements with baseline values within the same group. Normally distributed parametric data were presented as mean ± standard deviation (mean ± SD) and non-parametric data as median (minimum-maximum) values. The chi-square test was used to compare categoric data between the groups. Parameters that were statistically significant at univariate analysis were included in multivariate logistic regression analysis of the dependent variable of ICU mortality. p<0.05 values were considered statistically significant.

## RESULTS

Out of a total of 1200 patient files, only 850 with complete data were included in the study, since 200 patients were aged <45, 95 were children, and 55 records could not be accessed or had missing data. Baseline characteristics of the patients are presented in Table-I.

**Table-I:** Demographics and characteristics of ICU patients (n=850)

Age, years (mean ± SD)	63±12
Female/male n (%)	407(48)/443(52)
Max admission HR (mean ± SD)	94±19
Min admission HR (mean ± SD)	68±142
Max HR after BB (mean ± SD)	79±13
Min HR after BB (mean ± SD)	69±10
Admission SBP (mean ± SD)	134±25
Admission DBP (mean ± SD)	71±15
Hypotension n (%)	621 (73)
Admission GCS n (%)	
3-7 n (%)	89 (10)
8-12 n (%)	14 (2)
13-15 n (%)	744 (87)
Postoperative	736(86)
Inotropsn (%)	65 (8)
Vasopressors n (%)	66 (8)
Anemia n (%)	496 (59)
Admission MV n (%)	99 (12)
Hospitalization MV n (%)	79 (9)
Length of ICU hospitalization (median[5-95])	3 [0-120]
Length of hospitalization (median[5-95])	9 [1-130]
ICU death n (%)	47 (5)
Post ICU death n (%) Hospital death n (%)	30 (3)
CHARLSON (ort ± SD)	3.5±1.7
APACHE II (ort ± SD)	9.3±9.2

BB: beta blocker; HR: heart rate; ICU: intensive care unit; SD: standard deviation; SBP: systolic blood pressure; DBP: diastolic blood pressure; GKS: Glasgow Coma Scale; MV: mechanical ventilation; max: maximum; min: minimum.

The data from the two groups, in which the patients with maximum HR<100/min within 24 hour of ICU admission were compared with those with higher maximum HR within 24 hour of ICU admission, are presented in Table-II. Maximum HR value on admission was 83±11/min in Group I and 115±14/ minute in Group II (p=0.002). The use of vasopressor and inotropic drugs was higher in Group II than in Group I (p<0.001). Anemia and mechanical ventilation were more common in Group II than in Group I (p<0.005). Patients in Group II also had higher CHARLSON and APACHE-II scores and therefore stayed longer in ICU and in hospital and had higher ICU and hospital mortality compared to Group I (p<0.05).

**Table-II:** The comparison of the patients regarding maximum heart rates(<100, ≥100) within 24 hours of ICU admission data is presented as mean ± SD, median[5-95 confidence interval] or frequency (%).

	HR<100 in ICU n=563	HR≥100 in ICU n=287	P
Age, years (mean±SD)	62±10	63±13	0.004*
Female/Male n (%)	306(54)/257(46)	85(15)/202(36)	0.040*
Max admission HR (mean±SD)	83±11	115±14	0.002*
Min admission HR (mean±SD)	62±10	79±14	0.026*
Admission SBP (mean±SD)	135±22	133±29	0.439
Admission DBP (mean±SD)	70±14	72±17	0.219
Admission MAP (mean±SD)	94±18	94±21	0.902
Hypotension n (%)	407(72)	214(74)	0.280
Previous beta blocker use n (%)	85(15)	46(35)	0.397
Beta blocker use in the ICU n (%)	204(36)	98(34)	0.300
Admission GCS n (%)			
3-7 n(%)	37(7)	53(18)	0.900
8-12 n(%)	6(1)	9(3)	
13-15 n(%)	520(92)	224(78)	
Inotrops n (%)	26(5)	39(14)	p<0.001*
Vasopressors n (%)	26(5)	40(14)	p<0.001*
Anemia n (%)	310(55)	186(65)	0.004*
Admission MV n (%)	38(7)	61(21)	p<0.001*
Hospitalization MV n (%)	30(5)	49(17)	p<0.001*
Length of ICU hospitalization (median[5-95])	1[1-13]	p<0.05* 1[1-8]	
Length of hospitalization (median [5-95])	2[1-32]	9[1-57]	p<0.05*
Mortality in ICU n (%)	17(3)	30(10)	p<0.001*
Mortality after ICU (%)	11(2)	8(3)	0.291
Total mortality+ n (%)	28(5)	38(13)	p<0.001*
CHARLSON (mean±SD)	3.3±1.7	3.7±1.8	p<0.05*
APACHE II (mean±SD)	8.3±8.1	11.6±11.04	p<0.05*

BB: beta blocker; HR: heart rate; ICU: intensive care unit; SD: standard deviation; SBP: systolic blood pressure, DBP: diastolic blood pressure; GKS:Glasgow Coma Scale; MV: mechanical ventilation; max: maximum; min: minimum.

At multivariate backward stepwise analysis, in terms of the dependent variable of mortality in the ICU, max HR <100/ minute was identified as one of the two statistically independent variables among age, previous beta blocker use, beta blocker use in the ICU, hypotension, and CHARLSON and APACHE-II scores. (HR<100/min [Group I] β=-0.860 SE=0.436 Wald=3.893 Sig=0.048) (Table-III).

**Table-III:** Variables remaining in the equation of multivariate logistic regression analysis on the dependent variable of ICU mortality.

	B	S.E	Wald	Sig.
APACHE II	-0.223	0.026	76.234	0.000
HR<100 (Group-I)	-0.860	0.436	3.893	0.048
Constant	8.722	1.052	68.770	0.000

HR: heart rate; ICU: intensive care unit; On the dependent variable of mortality in ICU, max HR <100/min was found to be one of the two statistically independent variables among age, previous BB use, BB use in ICU, hypotension, and CHARLSON and APACHE-II scores variables

Comparison of the two groups based on beta blocker use revealed no statistically significant differences in terms of age, gender, use of inotropic and vasopressor drugs, length of ICU and hospital stay, mortality in the ICU, total mortality or CHARLSON and APACHE-II scores (Table-IV). Patients who used beta-blockers had higher SBP and DBP and more acceptable MAP values compared with patients who did not use beta-blockers. In contrast, the patients who did not use beta-blockers had higher rates of hypotension, anemia and MV application than the patients who used beta blockers (p <0.05).

**Table-IV:** The Comparison of the patients who used and didn’t use beta blockers in the ICU Data is presented as mean ± SD, median[5-95 confidence intervals] or frequency (%)

	Patients who used beta blockers in ICU n=302	Patients who didn’t use beta blockers in ICU n=548	P
Age, years (mean±SD)	64±12	62±11	0.168
Female/Male n(%)	152(51)/150(49)	255(46)/293(53)	0.161
Max admission HR(mean±SD)	94±18	94±20	0.871
Min admission HR (mean±SD)	68±14	68±15	0.351
Admission SBP (mean±SD)	141±25	131±24	<0.001*
Admission DKB (mean±SD)	73±15	70±16	0.007*
Admission MAP (mean±SD)	98±19	92±19	<0.001*
Hypotension n(%)	201(66)	420(77)	<0.001*
*Admission GCS n(%)*			
3-7 n(%)	22(7)	67(12)	0.058
8-12 n(%)	6(2)	8(2)	
13-15 n(%)	273(90)	471(85)	
Inotrops n(%)	21(7)	44(8)	0.337
Vasopressors n(%)	18(6)	48(9)	0.091
Anemia n(%)	234(78)	262(48)	<0.001*
Admission MV n(%)	21(7)	78(14)	0.001*
Hospitalization MV n(%)	20(7)	59(9)	0.029*
Length of ICU hospitalization (median[5-95])	1[0-63]	1[1-120]	0.117
Length of hospitalization (median[5-95])	2[1-80]	3[1-130]	0.151
ICU death n(%)	12(4)	35(16)	0.092
Post ICU death n(%) Hospital death n(%)	9(3) 21(7)	21(4) 56(20)	0.39 0.06
CHARLSON (mean±SD)	3,6±1,7	3,4±1,7	0.187
APACHE II (mean±SD)	8,8±8,6	9,57±9,4	0.858

* p<0.05; MV: mechanical ventilator; ICU: intensive care unit; SD: standard deviation;

HR: heart rate; SBP: systolic blood pressure, DBP: diastolic blood pressure; GCS: Glasgow Coma Scale; MAP: mean arterial pressure; max: maximum; min: minimum.

## DISCUSSION

The results of our study demonstrate that maximum HR<100/minutes within 24 hours of ICU admission is an independent predictor of ICU mortality, independently of disease severity in a mixed ICU patient population. This retrospective analysis including 850 medical and surgical ICU patients supported the hypothesis that maximum HR<100/min would be associated with better outcomes in the ICU. Although severity of disease has a direct role predicting mortality, the clinical usefulness of controlling maximum HR within 24 hours of admission as a prophylactic general measure in ICU may reduce mortality.

Following the publication of guidelines supporting beta blocker use in high risk patients undergoing non-cardiac surgery and using beta blockers in many different clinical settings, pre-ICU practitioners and intensivists have become more HR-conscious, and their use has increased.

In our study, patients who used beta blockers before admission to the ICU did not achieve target HRs. Although the use of beta blockers reduced both resting mean and maximal HR in response to exercise and hypoxia, no significant differences were observed in patients using beta blockers in either group. Patients who used beta-blockers had higher SBP and DBP and more acceptable MAP values than those who did not use beta-blockers. In contrast, patients who did not use beta-blockers had higher rates of hypotension, anemia and MV application than those who used beta blockers (p <0.05). This reflected the limitations of indications for beta blockers, rather than their effects. This showed that beta blockers were not used in some patients because of hypotension, anemia and MV application.

In acute illness, HR generally reflects the severity of the underlying disease and is regarded as a risk marker. Beattie et al. analyzed HR responses and defined the maximal HR as the upper range of HR and assessed the effect of HR on the heterogeneity described in a previous meta-analysis. They reported a significant reduction in the incidence of postoperative myocardial infarction in trials in which patients receiving beta blockers had a maximal HR of <100/min, whereas in trials where patients had maximal HR greater than 100/min there was no reduction in MI.^10^

Nolte et al. found an independent association between the highest quartile of HR on admission (>86 bpm) and any deaths, exacerbation of heart failure and a higher degree of dependence in acute stroke patients without atrial fibrillation.^13^ Also, previous studies have shown associations between increased heart rate and cardiovascular risk in various populations. In a study, Lonn et al. examined and examined associations between heart rate and cardiovascular events in a contemporary medically optimized population with stable cardiovascular disease. Resting and, in particular, in-trial average heart rate are independently associated with significant increases in cardiovascular events and all-cause death.^14^

Erdur et al. investigated the association between HR measured on admission and early in-hospital mortality in acute ischemic stroke patients. Higher HR on admission was associated with a higher risk for in hospital mortality in age-adjusted logistic regression analysis. Especially patients with HR ≥83 bpm (highest tertile)showed a higher risk for in-hospital mortality compared to the reference tertile (≤69 bpm). HR remained significantly associated with in-hospital mortality in a multiple logistic regression analysis adjusted for age, stroke severity, congestive heart failure, and development of pneumonia.^15^

Our group consisted mainly of postoperative patients who had undergone non-cardiac surgery. In our study, we investigated the threshold limit within 24 hours of admission in our all ICU patients and showed that maximal HR<100/min at admission was independently associated with decreased ICU mortality. A limit that can be employed generally in mixed ICU patients is practical, yet 100/min is a very commonly used threshold.

### Limitations of this study

These should be taken into consideration when interpreting the findings. First, it is a retrospective and single-center study. The confounding drug history of some patients should also have been evaluated. However, no detailed drug history was available in some cases. Our population consisted of mostly hypotensive, postoperative, anemic patients with relatively lower APACHE-II scores with short ICU stays, all of which may influence the general applicability of these results.

The strength and primary utility of this analysis is the generation of hypotheses to guide future research concerning the relationship between the tight HR control with beta blockers within 24 hours of admission and mortality in the ICU. Several issues must be addressed in order to achieve adequate control of HR, as this is problematic in many patients. The reasons for inadequate HR are multifactorial and need to be investigated by further studies.

### Conclusion

HR<100/minutes within 24 hours of admission to a mixed ICU was correlated with ICU mortality. Although severity of diseases is directly involved in predicting mortality, further studies should be conducted to determine whether controlling maximum HR as a prophylactic general procedure in the ICU can reduce mortality.

## References

[1] Bruce TA, Chapman CP, Baker O, Fisher JN (1963). Role of autonomic and myocardial factors in cardiac control. J Clin Invest.

[2] Jouven X, Empana JP, Escolano S, Buyck JF, Tafflet M, Desnos M (2009). Relation of heart rate at rest and long-term (>20 years) death rate in initially healthy middle-aged men. Am J Cardiol.

[3] Hoke RS, Muller-Werdan U, Lautenschlager C, Werdan K, Ebelt H (2012). Heart rate as an independent risk factor in patients with multiple organ dysfunction: a prospective, observational study. Clin Res Cardiol.

[4] Kristal-Boneh E, Silber H, Harari G, Froom P (2000). The association of resting heart rate with cardiovascular, cancer and all cause mortality: eight year follow-up of 3527 male Israeli employees (the CORDIS Study). Eur Heart J.

[5] Booker KJ, Holm K, Drew BJ, Lanuza DM, Hicks FD, Carrigan T (2003). Frequency and outcomes of transient myocardial ischemia in critically ill adults admitted for non-cardiac conditions. Am J Crit Care.

[6] Marik PE, Marik PE (2001). Supraventricular and ventricular arrhythmias. Handbook of Evidence- Based Critical Care.

[7] Magder SA (2012). The ups and downs of heart rate. Crit Care Med.

[8] Copie X, Hnatkova K, Staunton A, Fei L, Camm AJ, Malik M (1996). Predictive power of increased heart rate versus depressed left ventricular ejection fraction and heart rate variability for risk stratification after myocardial infarction. Results of a two-year follow up study. J Am Coll Cardiol.

[9] Guarracino F, Tritapepe L (2011). The use of beta-blockers and the importance of heart rate control in the perioperative and surgical intensive care settings. Hot Topics Cardiol.

[10] Beattie WS, Wijeysunders DN, Karkouti K, McCluskey S, Tait G (2008). Does tight heart rate control improve beta-blocker efficacy? An updated analysis of the non-cardiac surgical randomized trials. AnesthAnalg.

[11] Kop WJ, Verdino RJ, Gottdiener JS, O’Leary ST, BaireyMerz CN, Krantz DS (2001). Changes in heart rate and heart rate variability before ambulatory ischemic events. J Am Coll Cardiol.

[12] Disegni E, Goldbourt U, Reicher-Reiss H, Kaplinsky E, Zion M, Boyko V (1995). The predictive value of admission heart rate on mortality in patients with acute myocardial infarction. SPRINT Study Group. Secondary Prevention Reinfarction Israeli Nifedipine Trial. J Clin Epidemiol.

[13] Nolte CH, Erdur H, Grittnerb U, Schneiderb A, Piperb SK, Scheitza JF (2014). Impact of heart rate on admission on mortality and morbidity in acute ischaemic stroke patients – results from VISTA. Euro J Neurol.

[14] Lonn E, Rambihar S, Gao P, Custodis F, Sliwa K, Teo K (2016). Heart rate is associated with increased risk of major cardiovascular events, cardiovascular and all-cause death in patients with stable chronic cardiovascular disease: an analysis of ONTARGET/TRANSCEND. Clin Res Cardiol.

[15] Erdur H, Grittner U, Scheitz JF, Laufs U, Endres M, Nolte CH (2014). Heart rate on admission independently predicts in-hospital mortality in acute ischemic stroke patients. Int J Cardiol.

